# Interplay between Beryllium Bonds and Anion-π Interactions in BeR_2_:C_6_X_6_:Y^−^ Complexes (R = H, F and Cl, X = H and F, and Y = Cl and Br)

**DOI:** 10.3390/molecules20069961

**Published:** 2015-05-29

**Authors:** Marta Marín-Luna, Ibon Alkorta, José Elguero, Otilia Mó, Manuel Yáñez

**Affiliations:** 1 Instituto de Química Médica (CSIC), Juan de la Cierva, 3, 28006-Madrid, Spain; 2 Departamento de Química, Módulo 13, Universidad Autónoma de Madrid, Campus de Excelencia UAM-CSIC, Cantoblanco, E-28049 Madrid, Spain

**Keywords:** beryllium-π interactions, anion-π interactions, *ab initio* calculations, cooperativity

## Abstract

A theoretical study of the beryllium bonds in BeR_2_:C_6_X_6_ (R = H, F, Cl and X = H and F) has been carried out by means of MP2/aug′-cc-pVDZ computational methods. In addition, the ternary complexes BeR_2_:C_6_X_6_:Y^−^ (Y = Cl and Br) have been analyzed. Geometric, energetic and electronic aspects of the complexes have been taken into account. All the parameters analyzed provide a clear indication of favorable cooperativity in both interactions observed, beryllium bond and aromatic ring:anion interaction.

## 1. Introduction

In 2002, three independent groups showed theoretically for the first time the possibility of finding attractive anion-π interactions when the π system is electron deficient [1,2,3], hexafluorobenzene being a paradigmatic case. These theoretical calculations were supported by crystallographic data found in the Cambridge Structural Database (CSD) [1,4]. It was suggested that this novel mode of bonding could be used for developing new receptors for the recognition of anions [2]. Relationships have been found between the aromaticity of perfluoroaromatic compounds and their relative interaction energy with anions [3]. Since then, the number of papers reporting anion-π interactions has become very large; the reader can consult some reviews or very general papers [5,6,7] and two books [8,9]. Particularly informative is an experimental paper by Wang and Wang [10] based on 1,3,5-triazine, another of the classical π-deficient systems [2]. Other experimental papers reported solution studies [11] and crystallographic structures [12], both based on the C_6_F_5_ substituent.

Somewhat related to the anion-π interactions topic is the use of aromatic compounds as charge insulators. Many examples have been reported: Na^+^:C_6_H_6_:F^−^ and Na^+^:C_6_F_6_:F^−^ [13]; Li^+^:C_6_H_6_: F^−^; K^+^:C_6_F_6_:Br^−^ [14]; M^+^:C_6_H_3_F_3_:C_6_H_3_F_3_:X^−^ [15]; M^+^:C_6_F_6_:Cr:C_6_H_6_:X^−^ [16]; M^+^:C_6_H_6_:C_6_F_6_:X^−^ [17]; cyclopropenyl^+^:C_6_H_6_:phenalenyl^−^ [18]; Na^+^:1,3,5-triethynylbenzene: Cl^−^ [19]; Li^+^:C_6_R_6_:F^−^, R = H, F, Cl, Br, OMe [20], and −Na^+^:C_6_H_3_F_3_:Cl^−^ [21]. These have been extended to other insulators like hexafluoroethane [Na^+^:C_2_F_6_:Cl^−^] [22], saturated cycloalkanes like Li^+^:adamantane:F^−^ [23], cationic complexes like ZY_4_^+^:C_6_R_6_:YX, example: NH_4_^+^:C_6_H_6_:HF [24] as well as anionic complexes as XH:C_2_F_4_:Y^−^ [25].

Among the new non-covalent interactions discovered in the last years, beryllium bonds provide very strong complexes [26] and significantly alter the properties of the bonded systems [27,28,29,30,31,32,33,34]. Recently it has been shown that beryllium derivatives can interact with π-systems, such as ethylene or acetylene, to yield rather stable complexes [35]. In the present paper we will explore the structure and stability of the complexes of BeR_2_ derivatives with benzene, as the aromatic reference system, and with its hexafluoro derivative, C_6_F_6_, which should behave as a much weaker Lewis base than the parent C_6_H_6_. The second part of the paper will be devoted to analyze the similarities and dissimilarities between the complexes formed between these two aromatic compounds and halogen anions, namely Cl^−^ and Br^−^. In the third part we will analyze the effect of the simultaneous interaction of beryllium derivatives and halogen anions with benzene and hexafluorobenzene. A comparison between the binary complexes studied in the first two parts of the paper and the triads contemplated in the third part will allow us to detect possible cooperative effects between both kinds of non-covalent interactions within the triads.

## 2. Computational Methods

The geometry of the systems has been fully optimized with the MP2 computational method [36] and the aug′-cc-pVDZ basis set. This basis set corresponds to the aug-cc-pVDZ [37] one for the heavy atoms and to the cc-pVDZ one for the hydrogens. Frequency calculations have been carried out at the same computational level to confirm that the structures obtained correspond to energetic minima. All these calculations have been carried out with the Gaussian-09 program [38].

The many-body interaction-energy formalism (MBIE) [39,40] has been applied to obtain one-, two- and three-body contributions to the binding energy. For a ternary complex, the binding energy ∆E can be decomposed into one- (Equation (2)), two- (Equation (3)), and three-body interactions (Equation (4)), as:

(1) $$\Delta E=E(ABC)-\sum_{i=A}^{C}E_{m}(i)=\text{​}\sum_{i=A}^{C}\left[E(i)-E_{m}(i)\right]+\sum_{i=A}^{B}\sum_{j>i}^{C}\Delta^{2}E(ij)+\Delta^{3}E(ABC)$$

(2) $$E_{R}(i)=\text{​}E(i)-E_{m}(i)$$

(3) $$\Delta^{2}E(ij)=E(ij)-\left[E(i)-E(j)\right]$$

(4) $$\Delta^{3}E(ABC)=E(ABC)-\left[E(A)+E(B)+E(C)\right]-\left[\Delta^{2}E(AB)+\Delta^{2}E(AC)+\Delta^{2}E(BC)\right]$$

*E*_m_(*i*) is the energy of an isolated, optimized monomer, while *E*(*i*) is the monomer energy at its geometry in the complex. *E_R_*(*i*) is the monomer distortion energy. Δ^2^*E*(*ij*) and Δ^3^*E*(*ABC*) are the two- and three-body interaction energies computed at the corresponding geometries in the complex.

The topological analysis of the electron density of the systems has been carried out within the framework of the Atoms in Molecules (AIM) [41,42] methodology with the AIMAll [43] program using the MP2/aug′-cc-pVDZ wavefunction. The electronic properties and charge transfer of the complexes have been analyzed with the NBO method [44] using the NBO 3.1 program [45] at the B3LYP/aug′-cc-pVDZ//MP2/aug′-cc-pVDZ computational level.

The effect of the complexation on the aromaticity of benzene and hexaflurobenzene has been calculated by means of the HOMA index (Equation (5)) [46]. The value of the C-C bond length (1.408 Å) obtained for the isolated benzene at MP2/aug′-cc-pVDZ level has been used as *R_opt_* and for the value of α for C-C bonds the reported value has been used [47].

(5) $$HOMA=1-\frac{1}{n}\sum_{j=1}^{n}\alpha {\left(R_{opt}-R_{j}\right)}^{2}$$

## 3. Results and Discussion 

This section has been divided in four parts. In the first part, a brief mention to the electronic properties of the isolated benzene and hexafluorobenzene will be considered. In the second and third parts, the BeR_2_:C_6_X_6_ and C_6_X_6_:Y^−^ binary complexes will be respectively discussed. Finally, the last part will be devoted to the ternary BeR_2_:C_6_X_6_:Y^−^ complexes. The geometry, energy and molecular graphs of all the systems studied in the present article can be found in Tables S1 and S2 of the Supplementary Materials.

### 3.1. C_6_X_6_ Isolated Monomers

**Figure 1:**
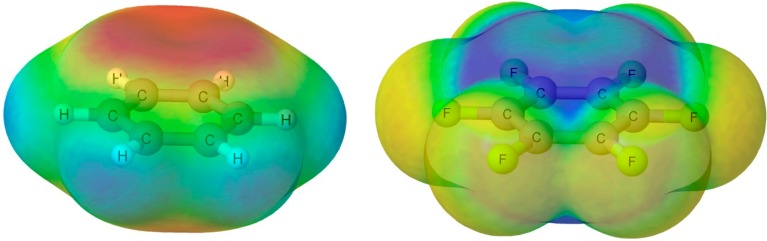
Electrostatic potential on the 0.001 au electron density of the isolated C_6_H_6_ (**left**) and C_6_F_6_ (**right**). The most intense red and blue color regions correspond to the −0.02 and +0.03 au values, respectively.

The electrostatic properties of the benzene and hexafluorobenzene molecules have been already discussed several times in the literature, especially in the context of their different tendency to form π-complexes [48]. Thus, benzene shows negative values of the electrostatic potential above and below the aromatic ring and tends to form complexes with positively charged groups or hydrogen bond donors [49,50,51,52,53]. In contrast, the electrostatic potential of the C_6_F_6_ molecule in both sides of the molecular plane presents positive values and consequently tends to form complexes with electron rich groups or anions [48,54]. The differences in the electrostatic potential of these two molecules have been rationalized based on their quadrupole moment [13,55] (Figure 1).

### 3.2. BeR_2_:C_6_X_6_ Binary Complexes

The binding energy and intermolecular distances of BeR_2_:C_6_X_6_ complexes are listed in Table 1. The binding energies of the complexes with benzene range between −26 kJ/mol and −47 kJ/mol; the BeCl_2_ and BeH_2_ complexes are the most stable and the least stable, respectively. The binding energies for the C_6_F_6_ range between −13 kJ/mol and −25 kJ/mol and are about half of the analogous ones with C_6_H_6_.

**Table 1:** Binding energies (kJ/mol), intermolecular distances (Å) and R-Be-R bond angle (°) of the BeR_2_:C_6_X_6_ binary complexes.

System	Eb	Be···Z*	> R-Be-R	System	Eb	Be···Z*	> R-Be-R
BeH_2_:C_6_H_6_	−25.7	2.575	157.5	BeH_2_:C_6_F_6_	−13.1	2.945	179.0
BeF_2_:C_6_H_6_	−41.4	2.214	146.4	BeF_2_:C_6_F_6_	−15.8	2.916	178.6
BeCl_2_:C_6_H_6_	−46.7	2.182	139.7	BeCl_2_:C_6_F_6_	−24.6	3.213	177.7

Z* represents the middle of the closest C-C bond of the aromatic system.

**Figure 2:**
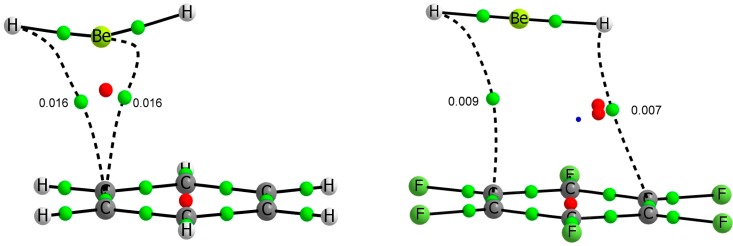

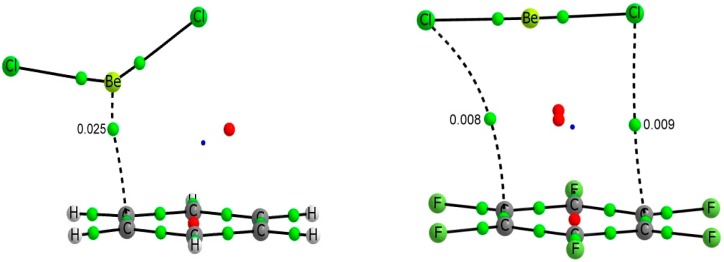
Molecular graph of BeR_2_:C_6_H_6_ (R = H, Cl) (**left**) and BeR_2_:C_6_F_6_ (R = H, Cl) (**right**) binary complexes. Green, red and blue dots denote BCPs, ring critical points and cage critical points respectively. The value of the electron density at the intermolecular BCP is indicated.

The molecular graph of the BeCl_2_:C_6_H_6_ and BeCl_2_:C_6_F_6_ complexes have been represented in Figure 2, as a suitable case for BeR_2_:C_6_H_6_ and BeR_2_:C_6_F_6_ systems. Clear differences are observed between the two families of complexes. In complexes with C_6_H_6_, the beryllium atom of the BeR_2_ derivatives is located above and close to one of the C-C bonds and slightly out of the aromatic ring while in the C_6_F_6_ family the Be is far from the C-C bond and placed close to the center of the aromatic ring.

The NBO analysis offers some clue on the origin of the aforementioned differences between BeR_2_:C_6_H_6_ and BeR_2_:C_6_F_6_ complexes. In both cases the aromatic moiety behaves as a Lewis base with respect to the BeR_2_ moiety, since a clear charge donation from the occupied π_cc_ orbitals of the aromatic into the empty p orbitals of Be and into the σ_BeR_* antibonding orbital is detected from the calculated second order orbital perturbation energies. The former are responsible for the bending undergone by the BeR_2_ moiety and the latter for the lengthening of the Be-R distances when BeR_2_ forms part of the complex. The NBO analysis shows that for C_6_H_6_ complexes, the larger contribution comes from a couple of C=C bonds, reflecting that the orbital interaction energies strongly depend on the overlap of the interacting occupied and empty orbitals. Clearly, the specific interaction with two of the CC bonds is privileged with respect to an equal interaction with the six bonds because in the first situation the overlap is much more efficient. In the case of the C_6_F_6_, the aforementioned interactions are much weaker, since C_6_F_6_ is a much poorer electron donor than C_6_H_6_. Indeed, as indicated in Table 2, the natural charges obtained within the NBO approach clearly show that the charge transfer from the aromatic systems towards the beryllium derivatives, is about three times larger when the aromatic is benzene than when it is C_6_F_6_.

**Table 2:** NBO charges (e) of the aromatic system within the BeR_2_:C_6_X_6_ complexes.

	NBO Charges (e)		NBO Charges (e)
BeH_2_:C_6_H_6_	0.048	BeH_2_:C_6_F_6_	0.017
BeF_2_:C_6_H_6_	0.066	BeF_2_:C_6_F_6_	0.005
BeCl_2_:C_6_H_6_	0.116	BeCl_2_:C_6_F_6_	0.012

However, also in C_6_F_6_ complexes there is a tendency to privilege the donation for only one couple of CC bonds. Actually, as shown in Figure 2, the BeR_2_ moiety does not sit strictly above the center of the ring, but it is also slightly displaced towards one of its CC bonds. However, since the interactions for C_6_F_6_ are much weaker than for benzene, the distance between both moieties is much longer, and the overlap does not privilege significantly the interaction with a specific pair of CC bonds, with respect to the others, leading to a more centered position of the BeR_2_ subunit. The fact that C_6_F_6_ is a much poorer electron donor than C_6_H_6_ is also clearly mirrored on the fact that in the C_6_H_6_ complex, the disposition of the three atoms of the BeR_2_ molecule is far from linearity, reaching R-Be-R angles of 140° in the strongest complex, while in the complexes with C_6_F_6_ the change of this angle is very small (less than 2.5°).

It is worth noting that the BeR_2_:C_6_F_6_ complexes with the beryllium atom along the *C*_6_ symmetry axes, which have a *C*_2*v*_ symmetry, present one imaginary frequency and a very small relative energy (less than 2.0 kJ/mol) with respect to the equilibrium conformation, corresponding to a transition state between two identical structures.

In line with the NBO analysis discussed above, the AIM approach shows the existence of just one intermolecular BCP between the beryllium atom and the centre of a C-C bond for complexes involving benzene (Figure 2). The values of the electron density at these BCPs range between 0.016 (BeH_2_) and 0.025 au (BeCl_2_). Positive values of the Laplacian and negative total energy density (between −0.003 and −0.006 au) are found in the BCPs (see Table 3), confirming that these interactions have a certain covalent character [56].

In the BeR_2_:C_6_F_6_ complexes, mentioned above, the interaction is much weaker and more delocalized, the intermolecular BCPs link the R atoms with the aromatic ring through two opposite C-C bonds. The electron density at the BCPs is rather small (between 0.009 and 0.007 au) and the Laplacian and total energy density are positive or nearly zero (Table 3).

**Table 3:** AIM parameters (in au) for the BCPs corresponding to the intermolecular interactions in the BeR_2_:C_6_X_6_ binary systems, the electron density, ρ_BCP_, its Laplacian, ∇^2^ρ_BCP_, and the total electron energy density, H_BCP_.

System	ρ_BCP_	∇^2^ρ_BCP_	H_BCP_	Interaction
BeH_2_:C_6_H_6_	0.0157	0.0184	-0.0028	Be···π
BeF_2_:C_6_H_6_	0.0218	0.0409	-0.0052	Be···π
BeCl_2_:C_6_H_6_	0.0247	0.0577	-0.0059	Be···π
BeH_2_:C_6_F_6_	0.0085	0.0153	-0.0001	H···π
	0.0067	0.0192	0.0008	H···π
BeF_2_:C_6_F_6_	0.0091	0.0263	0.0008	F···π
	0.0084	0.0320	0.0012	F···π
BeCl_2_:C_6_F_6_	0.0079	0.0180	0.0004	Cl···π
	0.0085	0.0240	0.0008	Cl···π

The calculated HOMA aromaticity indexes for these complexes (See Table S3 of the Supplementary Materials) are very similar to the corresponding isolated aromatic molecules, being the largest differences 0.01 units.

The application of the MBIE partition method shows that for both families of compounds the distortion energy of the aromatic ring is very small, as it is also for the BeR_2_ systems in the complexes with C_6_F_6_ (See Table 4). In contrast, the distortion energies of the BeR_2_ molecules in the complexes with C_6_H_6_ present values between 11 and 39 kJ/mol in agreement with the geometrical perturbation already discussed. Consequently, the interaction energy (Δ^2^E) of these complexes reaches values up to −87 kJ/mol in the C_6_H_6_:BeCl_2_ case while in the ones with C_6_F_6_ the values of Δ^2^E are about four times smaller and very similar to those of the binding energies.

**Table 4:** Many body Interaction energy (MBIE) partition terms (kJ/mol) in the BeR_2_:C_6_X_6_ binary systems.

System	Er(Ar)	Er(BeR_2_)	Δ^2^E(BeR_2_:C_6_H_6_)	System	Er(Ar)	Er(BeR_2_)	Δ^2^E(BeR_2_:C_6_F_6_)
BeH_2_:C_6_H_6_	0.2	10.7	−36.6	BeH_2_:C_6_F_6_	0.16	0.03	−13.3
BeF_2_:C_6_H_6_	0.5	26.3	−68.2	BeF_2_:C_6_F_6_	0.3	0.1	−16.2
BeCl_2_:C_6_H_6_	0.9	39.0	−86.6	BeCl_2_:C_6_F_6_	0.3	0.05	−24.9

### 3.3. C_6_X_6_:Y^−^ Binary Complexes

**Figure 3:**
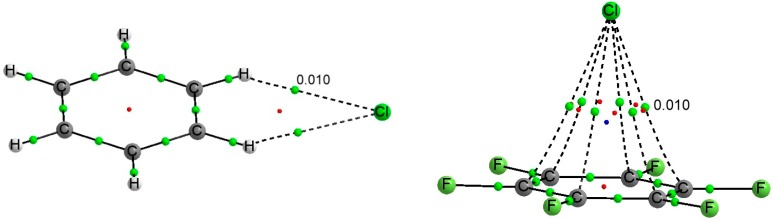
Molecular graph of the C_6_H_6_:Cl^−^ (**left**) and C_6_F_6_:Cl^−^ (**right**) complexes. Green, red and blue dots denote BCPs, ring and cage critical points respectively. The value of the electron density at the intermolecular BCP is indicated.

As expected from the characteristics of the molecular electrostatic potential discussed above, the equilibrium structure for C_6_H_6_:Y^−^ complexes is totally different from that of C_6_F_6_, in agreement with previous reports [3,57,58,59]. In the C_6_H_6_ complexes, the anion is located in the molecular plane, interacting simultaneously with two hydrogen atoms, whereas in the C_6_F_6_:Y^−^ complexes the anion sits on the *C_6_* symmetry axis and above the plane of the molecule. Figure 3 shows the molecular graph of two representative C_6_X_6_:Y^−^ complexes.

The binding energies of these complexes (Table 5) show that the C_6_F_6_:Y^−^ complexes are almost twice more stable than the C_6_H_6_:Y^−^ ones, in contrast with the results obtained for the BeR_2_:C_6_X_6_ complexes, simply because in the complexes with BeR_2_ the aromatic ring behaves as a Lewis base *versus* a rather strong Lewis acid, whereas in the complexes with Y^−^ they behave as a Lewis acid, which can only accept electrons in the π* antibonding orbitals. The nature of the halide has a small effect on the binding energy, the complexes with chloride being slightly more stable than with bromide. The MBIE partition (Table 4) shows very small distortion energies for the aromatic systems and consequently, the interaction energies (Δ^2^E) are very similar to the binding ones.

The molecular graph of these complexes (see Figure 3 for two examples) shows two degenerate Y···H BCPs in the C_6_H_6_:Y^−^ complexes, corresponding to the two hydrogen bonds between the halogen anion and the CH groups of benzene, and six Y···C BCP in C_6_F_6_:Y^−^. Those BCPs show similar values of the electron density, 0.010 au for the chloride complexes and 0.009 au for the bromide ones. In all cases, the BCPs show positive values of the Laplacian and total energy density.

**Table 5:** Binding energy (kJ/mol), intermolecular distance (Å), distortion energy and Δ^2^E (kJ/mol) in the C_6_X_6_:Y^−^ binary systems within the MBIE partition method.

System	Eb	Y···HC	Er(C_6_H_6_)	Δ^2^E(C_6_H_6_:Y)	System	Eb	Y···Z*	Er(C_6_F_6_)	Δ^2^E(C_6_F_6_:Y)
C_6_H_6_:Br^−^	−34.4	2.902	1.2	−35.6	C_6_F_6_:Br^−^	−65.8	3.433	0.7	−66.6
C_6_H_6_:Cl^−^	−35.9	2.743	1.6	−37.5	C_6_F_6_:Cl^−^	−67.1	3.290	0.9	−67.9

Z* represents the middle of one of the C-C bonds of the aromatic system.

The NBO analysis indicates a larger charge transfer for the C_6_H_6_:Y^−^ complexes (−0.026 and −0.027 e, for Y = Br and Cl, respectively) than for the C_6_F_6_:Y^−^ ones (−0.013 and −0.012 e), as a consequence of the rather different nature of both kinds of interactions, since, as indicated above the former are stabilized through intermolecular C-H···Y^−^ hydrogen bonds and the latter through Y^−^-π interactions. Coherently, the second order perturbation analysis indicates a charge transfer in the C_6_H_6_:Y^−^ complexes from the lone pairs of the anions towards the σ_CH_* antibonding orbitals with interaction energies up to 7.4 kJ/mol, while in the C_6_F_6_:Y^−^ ones, the expected charge transfer between the lone pair of the anions and the π_CC_* antibonding orbitals of the aromatic systems is very small (<0.7 kJ/mol).

### 3.4. BeR_2_:C_6_X_6_:Y^−^ Ternary Complexes

**Figure 4:**
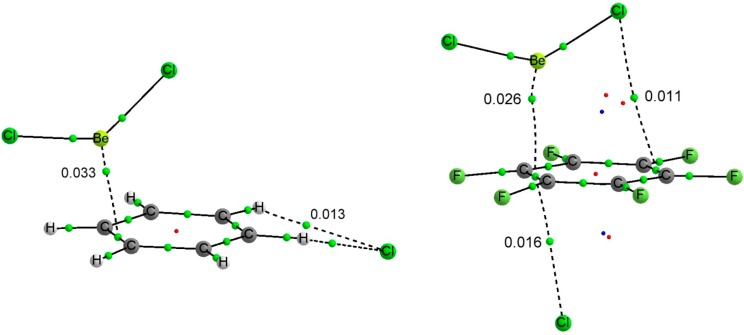
Molecular graph of BeCl_2_:C_6_H_6_:Cl^−^ (**left**) and BeCl_2_:C_6_F_6_:Cl^−^ (**right**). The value of the electron density at the intermolecular BCPs is indicated.

The binding energy and intermolecular distances of the BeR_2_:C_6_X_6_:Y^−^ (R = H, F, Cl; X = H, F; Y = Cl, Br) ternary complexes have been listed in Table 6. The molecular graphs of two representative ternary complexes have been represented in Figure 4.

**Table 6:** Binding energy (kJ/mol), intermolecular distances (Å) and R-Be-R bond angle (°) of the ternary complexes. The variations with respect to the corresponding binary complexes are also added.

System	Eb	Be···Z*	∆Be···Z*	Y···Z*	∆Y···Z*	∠ R-Be-R	∆∠ R-Be-R
BeH_2_:C_6_H_6_: Br^−^	−80.3	2.185	−0.390	2.820	−0.082	145.7	−11.8
BeH_2_:C_6_H_6_:Cl^−^	−83.2	2.177	−0.398	2.658	−0.085	145.2	−12.3
BeF_2_:C_6_H_6_:Br^−^	−104.6	2.089	−0.125	2.802	−0.100	138.1	−8.3
BeF_2_:C_6_H_6_:Cl^−^	−107.9	2.084	−0.130	2.639	−0.104	137.7	−8.7
BeCl_2_:C_6_H_6_ :Br^−^	−118.7	2.042	−0.140	2.778	−0.124	132.0	−7.7
BeCl_2_:C_6_H_6_:Cl^−^	−122.4	2.034	−0.148	2.616	−0.127	131.7	−8.0
BeH_2_:C_6_F_6_:Br^−^	−96.7	2.413	−0.532	3.204	−0.229	155.8	−23.2
BeH_2_:C_6_F_6_:Cl^−^	−99.0	2.396	−0.549	3.031	−0.259	154.8	−24.2
BeF_2_:C_6_F_6_:Br^−^	−112.8	2.270	−0.646	3.156	−0.277	144.6	−34.0
BeF_2_:C_6_F_6_:Cl^−^	−115.6	2.261	−0.655	2.990	−0.300	143.9	−34.7
BeCl_2_:C_6_F_6_:Br^−^	−122.5	2.253	−0.960	3.126	−0.307	137.9	−39.8
BeCl_2_:C_6_F_6_:Cl^−^	−125.8	2.242	−0.971	2.957	−0.333	137.2	−40.5

Z* represents the middle of the closest C-C bond of the aromatic system.

The binding energies in the ternary complexes range between −80 and −126 kJ/mol. The C_6_F_6_ complexes are always more stable than the analogous with C_6_H_6_. As in the case of the binary complexes, the ranking based on the beryllium derivative is BeH_2_ > BeF_2_ > BeCl_2_ and the difference between the binding energy in the chloride and bromide complexes is small, the chloride complexes always being more stable than the bromide ones. An excellent linear correlation is obtained between the binding energies in the C_6_F_6_ *vs.* the C_6_H_6_ series (*R*^2^ = 0.999).

The geometrical parameters listed in Table 6 already provide some clues about the cooperativity in the ternary complexes. The intermolecular distances between the aromatic systems and the beryllium derivatives are reduced up to 0.40 Å in the C_6_H_6_ series and up to 0.97 Å in the C_6_F_6_ ones when comparing to the corresponding binary complexes. In C_6_H_6_ complexes, the larger effects are observed for complexes with BeH_2_ and for the BeCl_2_ for C_6_F_6_ complexes. Similar shortening is observed for the intermolecular distances between the anions and the aromatic rings. The larger effect observed in both series corresponds to the complexes with BeCl_2_ being the calculated shortening 0.13 and 0.30 Å in the C_6_H_6_ and C_6_F_6_ series, respectively.

Another geometrical parameter that changes from the binary to the ternary complexes is the R-Be-R bond angle which is always smaller in the latter ones. The largest effect is observed in the BeCl_2_:C_6_F_6_:Y^−^ complexes, where the variation of the R-Be-R bond angle on going from the binary to the ternary complexes is 40°.

As in the case of the binary complexes, the calculated HOMA aromaticity indexes for the ternary complexes (See Table S3) are almost identical to those of the corresponding isolated aromatic molecules, being the largest differences 0.02 units.

The MBIE partition terms of the ternary complexes have been gathered in Table 7. The distortion energy in the aromatic molecules is small in all cases (between +1.7 and +4.3 kJ/mol), but larger than in binary complexes, while those of the beryllium derivatives complexed with C_6_H_6_ range between +26 and +59 kJ/mol and in the complexes with C_6_F_6_ between +12 and +44 kJ/mol, are also larger than in the binary complexes. The three Δ^2^E terms and the Δ^3^E one for all the compounds are negative. The largest stabilization energy is the Δ^2^E(BeR_2_:Ar) for the C_6_H_6_ complexes and Δ^2^E(Ar:Y) for the C_6_F_6_ ones. For the C_6_H_6_ complexes the second most important term is the Δ^2^E(Ar:Y) followed by the Δ^3^E(BeR_2_:Ar:Y) one, the least important one being the Δ^2^E(BeR_2_:Y). In the C_6_F_6_ complexes, Δ^2^E(BeR_2_:Y) is of similar magnitude to that of Δ^2^E(BeR_2_:Ar) in the BeR_2_:C_6_F_6_:Y for R = H and F while for R = Cl, Δ^2^E(BeR_2_:Ar) is more important than Δ^2^E(BeR_2_:Y). The negative value of ∆^3^E, which indicates strong cooperativity, ranges between −21 and −35 kJ/mol in the C_6_H_6_ complexes and between −13 and −24 kJ/mol in the C_6_F_6_ ones. The Δ^2^E(BeR_2_:Ar) term is always larger in absolute value in the ternary complexes than in the binary ones while the Δ^2^E(Ar:Y) one is slightly smaller in absolute value in the ternary than in the corresponding binary complexes.

**Table 7:** Many body Interaction energy (MBIE) partition term (kJ/mol) in the ternary systems *.

System	Er(Ar)	Er(BeR_2_)	Δ^2^E(BeR_2_:Ar)	Δ^2^E(Ar:Y)	Δ^2^E(BeR_2_:Y)	Δ^3^E(BeR_2_:Ar:Y)
BeH_2_:C_6_H_6_: Br^−^	2.1	25.8	−48.2	−34.5	−4.3	−21.1
BeH_2_:C_6_H_6_:Cl^−^	2.6	26.5	−48.7	−36.5	−4.5	−22.6
BeF_2_:C_6_H_6_:Br^−^	2.5	43.3	−81.7	−33.8	−11.0	−24.0
BeF_2_:C_6_H_6_:Cl^−^	3.0	44.3	−82.3	−35.8	−11.5	−25.6
BeCl_2_:C_6_H_6_ :Br^−^	3.4	58.4	−103.0	−33.1	−11.6	−32.8
BeCl_2_:C_6_H_6_:Cl^−^	4.0	59.3	−103.6	−35.1	−12.1	−34.8
BeH_2_:C_6_F_6_:Br^−^	1.7	12.4	−16.1	−65.2	−16.7	−12.9
BeH_2_:C_6_F_6_:Cl^−^	1.8	13.5	−16.3	−66.3	−18.3	−13.5
BeF_2_:C_6_F_6_:Br^−^	2.4	29.2	−32.7	−64.2	−32.3	−15.1
BeF_2_:C_6_F_6_:Cl^−^	2.4	30.4	−33.1	−65.2	−34.7	−15.4
BeCl_2_:C_6_F_6_:Br^−^	4.3	42.2	−49.5	−63.5	−33.0	−22.9
BeCl_2_:C_6_F_6_:Cl^−^	4.3	43.8	−50.2	−64.5	−35.5	−23.8

* The sum of these terms is equal to the binding energy.

The topology of the molecular graph of the BeR_2_:C_6_H_6_:Y^−^ complexes is similar to the sum of those of the corresponding dimers. However the electron density values in the intermolecular BCPs (Table 8) are larger in the ternary complexes than in the corresponding binary ones [0.033 vs. 0.0025 au in the Be-π BCP and 0.013 vs. 0.010 in the Cl···HC interaction in the BeCl_2_:C_6_H_6_:Cl^−^ complex and its corresponding binary complexes, Figure 2, Figure 3 and Figure 4] in agreement with the shorter intermolecular distances found in the former complexes and the relationship between the electron density at the BCP and the interatomic distance [60,61,62,63,64,65,66], and with the negative values of the ∆^3^E terms. As a consequence of the substantial reinforcement of both the beryllium bonds and the interaction between the aromatic and the anion Y^−^ on going from the binary complexes to the triads, the molecular graph of the triads BeR_2_:C_6_F_6_:Y^−^, presents a single intermolecular BCP between the anion and the aromatic ring (Figure 4) in contrast to the six BCPs found in the binary complexes (Figure 3) and a BCP connecting the beryllium atom with the aromatic ring while in the binary complexes the two BCPs were between the R groups and the aromatic ring. Consistently, for the BeR_2_:C_6_H_6_:Y^−^, both the electron density at the BCP connecting the beryllium atom with the aromatic ring and at the CH···Y^−^ hydrogen bonds are much larger in the triad than in the corresponding binary complexes.

**Table 8:** AIM parameters (in au) for the BCPs corresponding to the Be···π and π···Y interactions in the ternary systems, the electron density, ρ_BCP_, its Laplacian, ∇^2^ρ_BCP_, and the total electron energy density, H_BCP_.

System	Be···π			π···Y^−^		
	ρ_BCP_	∇^2^ρ_BCP_	H_BCP_	ρ_BCP_	∇^2^ρ_BCP_	H_BCP_
BeH_2_:C_6_H_6_: Br^−^	0.0224	0.0278	−0.0059	0.0111	0.0288	0.0005
BeH_2_:C_6_H_6_:Cl^−^	0.0227	0.0295	−0.0060	0.0123	0.0347	0.0007
BeF_2_:C_6_H_6_:Br^−^	0.0280	0.0860	−0.0048	0.0115	0.0299	0.0005
BeF_2_:C_6_H_6_:Cl^−^	0.0283	0.0872	−0.0048	0.0128	0.0362	0.0007
BeCl_2_:C_6_H_6_ :Br^−^	0.0326	0.0974	−0.0065	0.0120	0.0314	0.0005
BeCl_2_:C_6_H_6_:Cl^−^	0.0330	0.0990	−0.0070	0.0130	0.0380	0.001
BeH_2_:C_6_F_6_:Br^−^	0.0164	0.0182	−0.0041	0.0131	0.0364	0.0011
BeH_2_:C_6_F_6_:Cl^−^	0.0169	0.0190	−0.0043	0.0146	0.0455	0.0016
BeF_2_:C_6_F_6_:Br^−^	0.0222	0.0373	−0.0060	0.0140	0.0399	0.0012
BeF_2_:C_6_F_6_:Cl^−^	0.0226	0.0409	−0.0060	0.0155	0.0494	0.0017
BeCl_2_:C_6_F_6_:Br^−^	0.0252	0.0336	−0.0082	0.0147	0.0421	0.0012
BeCl_2_:C_6_F_6_:Cl^−^	0.0257	0.0377	−0.0082	0.0163	0.0524	0.0018

Similar reinforcements of both non-covalent interactions become evident when the NBO analysis is employed, reflected in much larger charge transfer towards the beryllium derivative from both the anion and the aromatic systems (Table 9). At the same time, the second order perturbation analysis shows an increment of the charge transferred from the C-C bonds of the aromatic systems towards the empty ones of the beryllium that corresponds to E(2) stabilization values of 98 and 21 kJ/mol in the BeH_2_:C_6_X_6_:Cl^−^, with X = H and F, respectively.

**Table 9:** Charge (e) of the monomers in the ternary complex.

System	Aromatic	BeR_2_	Y^−^
BeH_2_:C_6_H_6_: Br^−^	0.087	−0.124	−0.963
BeH_2_:C_6_H_6_:Cl^−^	0.088	−0.126	−0.962
BeF_2_:C_6_H_6_:Br^−^	0.058	−0.010	−0.959
BeF_2_:C_6_H_6_:Cl^−^	0.059	−0.101	−0.958
BeCl_2_:C_6_H_6_ :Br^−^	0.115	−0.161	−0.953
BeCl_2_:C_6_H_6_:Cl^−^	0.117	−0.163	−0.953
BeH_2_:C_6_F_6_:Br^−^	0.067	−0.104	−0.963
BeH_2_: C_6_F_6_:Cl^−^	0.070	−0.107	−0.962
BeF_2_: C_6_F_6_:Br^−^	0.116	−0.167	−0.949
BeF_2_: C_6_F_6_:Cl^−^	0.120	−0.172	−0.948
BeCl_2_: C_6_F_6_:Br^−^	0.060	−0.102	−0.958
BeCl_2_: C_6_F_6_:Cl^−^	0.063	−0.107	−0.957

## 4. Conclusions

Our MP2/aug′-cc-pVDZ theoretical survey of the complexes formed by two aromatic systems (C_6_H_6_ and C_6_F_6_) when interacting simultaneously with beryllium derivatives (BeH_2_, BeF_2_ and BeCl_2_) and anions (Cl^−^ and Br^−^) shows that the shape of the complexes depends on the aromatic ring. C_6_H_6_ yields complexes where the anions are practically lying in the molecular plane of the aromatic system, and are stabilized by CH···Y^−^ hydrogen bonds. Conversely, for C_6_F_6_ complexes, the Y^−^ anions are located along the *C*_6_ axis and above the ring to favor the interaction with the π electrons. The beryllium derivatives are close to one of the C-C bonds of the aromatic moiety in all the complexes (binary and ternary) with C_6_H_6_ while in the C_6_F_6_ binary complexes they are much farther away, due to the much smaller electron donor capacity of C_6_F_6_. Strong cooperative effects are found when comparing the interactions in the triads with those in the corresponding binary complexes. Indeed, the electronic density distribution of the BeR_2_:aromatic:Y^−^ ternary complexes reflects these cooperative effects by a significant increase of the electron density at the intermolecular BCPs between the beryllium derivative and the aromatic system and between the aromatic system and the Y^−^ anion. Also the MBIE analysis accounts for this cooperativity mirrored in significant negative values of the three-body interaction energy, Δ^3^E. Although these interactions have a clear electrostatic component, they also show significant polarization effects which lead to significant deformations of the BeR_2_ moiety, which becomes clearly bent with longer Be-R bonds, through a charge transfer to the empty p orbitals of Be and to the σ_BeR_* antibonding orbitals. This cooperativity is in agreement with the combination of π-anion contacts with other weak interactions (halogen and hydrogen bonds) already described in the literature [67].

## Supplementary Materials

Supplementary materials can be accessed at: http://www.mdpi.com/1420-3049/20/06/9961/s1.
