# Supplementary material for: Interplay between Beryllium Bonds and Anion-π Interactions in BeR2:C6X6:Y− Complexes (R = H, F and Cl, X = H and F, and Y = Cl and Br)
Source: Molecules. 2015 May 29;20(6):9961–76. doi: 10.3390/molecules20069961 (PMC6272305; doi:10.3390/molecules20069961)
Supplement: Supplementary file 1 [file molecules-20-09961-s001.pdf]

Supplementary Materials

Interplay between Beryllium Bonds and Anion-π Interactions in BeR_2:C_6X_6:Y^- Complexes (R = H, F and Cl, X = H and F, and Y = Cl and Br)

Table S1. Molecular graphs, MP2 energy (Hartree), Number of Imaginary Frequencies (NIMAG) and Cartesian coordinates (Å) of C_6H_6, C_6H_6: X^-, BeR_2:C_6H_6 and BeR_2:C_6H_6: X^- systems.

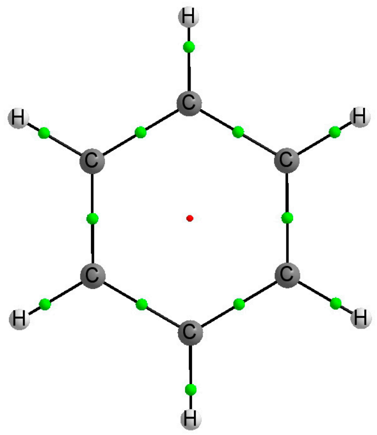	**C_6H_6** MP2 = −231.53535814 NIMAG = 0<br>C, 0.0000000, 0.0000000, 1.4080620<br>C, 1.2194175, 0.0000000, 0.7040310<br>C, 1.2194175, 0.0000000, −0.7040310<br>C, 0.0000000, 0.0000000, −1.4080620<br>C, −1.2194175, 0.0000000, −0.7040310<br>C, −1.2194175, 0.0000000, 0.7040310<br>H, 0.0000000, 0.0000000, 2.5030653<br>H, 2.1677182, 0.0000000, 1.2515327<br>H, 2.1677182, 0.0000000, −1.2515327<br>H, 0.0000000, 0.0000000, −2.5030653<br>H, −2.1677182, 0.0000000, −1.2515327<br>H, −2.1677182, 0.0000000, 1.2515327
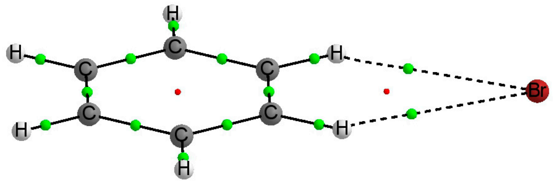	**C_6H_6:Br^-** MP2 = −2804.15776381 NIMAG = 0<br>C, −0.7043361781, 0., −2.5519491361<br>C, −1.4057425715, 0., −1.3285831579<br>C, −0.7045442698, 0., −0.1066306298<br>C, 0.7045442698, 0., −0.1066306298<br>C, 1.4057425715, 0., −1.3285831579<br>C, 0.7043361781, 0., −2.5519491361<br>H, −1.2524561776, 0., −3.5016246387<br>H, −2.502071949, 0., −1.33007136<br>H, −1.2144825587, 0., 0.863414021<br>H, 1.2144825587, 0., 0.863414021<br>H, 2.502071949, 0., −1.33007136<br>H, 1.2524561776, 0., −3.5016246387<br>Br, 0., 0., 3.4993293131

Table S1. *Cont.*

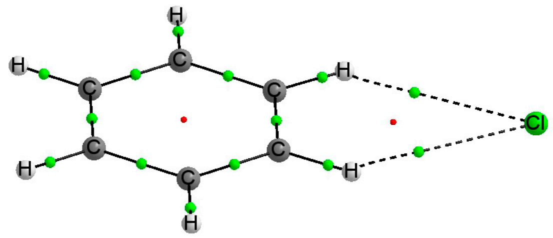	**C_6H_6:Cl^-** MP2 = −691.27180164 NIMAG = 0<br>C, −0.7043236309, 0., −2.5396581427<br>C, −1.4058468748, 0., −1.3158622779<br>C, −0.7046877532, 0., −0.0939338865<br>C, 0.7046877532, 0., −0.0939338865<br>C, 1.4058468748, 0., −1.3158622779<br>C, 0.7043236309, 0., −2.5396581427<br>H, −1.2529266375, 0., −3.4892075267<br>H, −2.5023228441, 0., −1.3179720035<br>H, −1.2082109439, 0., 0.8798521549<br>H, 1.2082109439, 0., 0.8798521549<br>H, 2.5023228441, 0., −1.3179720035<br>H, 1.2529266375, 0., −3.4892075267<br>Cl, 0., 0., 3.3420028749
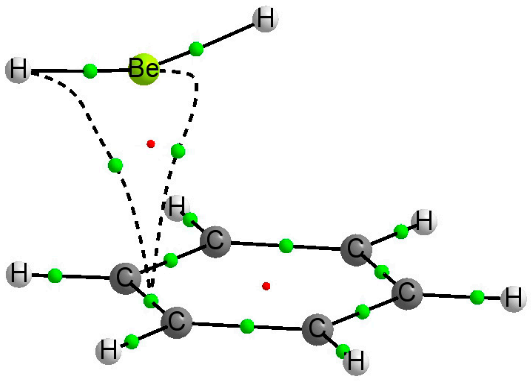	**C_6H_6:BeH_2** MP2 = −247.3635869 NIMAG = 0<br>C, 0.7521423406, −0.7647137121, 0.7015018414<br>C, 0.2112393693, 0.3212539413, 1.3998053119<br>C, −0.3261276451, 1.4046642969, 0.6987443107<br>C, −0.3261276451, 1.4046642969, −0.6987443107<br>C, 0.2112393693, 0.3212539413, −1.3998053119<br>C, 0.7521423406, −0.7647137121, −0.7015018414<br>H, 1.1882017202, −1.5996498902, 1.2420274688<br>H, 0.2115469181, 0.3218127011, 2.4858522813<br>H, −0.7494057432, 2.2448954459, 1.2412119946<br>H, −0.7494057432, 2.2448954459, −1.2412119946<br>H, 0.2115469181, 0.3218127011, −2.4858522813<br>H, 1.1882017202, −1.5996498902, −1.2420274688<br>Be, −1.4427582466, −2.2024622922, 0.<br>H, −0.8148440282, −3.3852293633, 0.<br>H, −2.3825565037, −1.2547419057, 0.

Table S1. *Cont.*

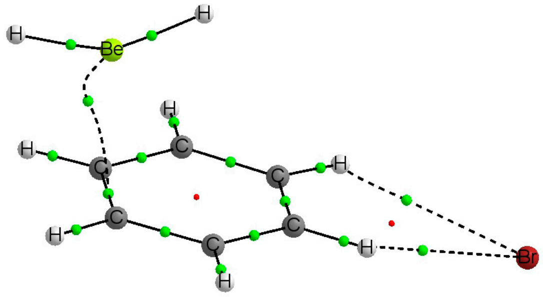

Br^-:C_6H_6:BeH_2 MP2 = −2819.99368763 NIMAG = 0
C, 0.6143364987, −1.1857977836, 0.7089752856
C, 0.3849145021, 0.019921447, 1.407666951
C, 0.1883244528, 1.2240942743, 0.7038536878
C, 0.1883244535, 1.224094274, −0.7038536885
C, 0.3849145036, 0.0199214463, −1.4076669509
C, 0.6143364995, −1.185797784, −0.7089752847
H, 0.8199460884, −2.1143749016, 1.2526227457
H, 0.380244482, 0.0171777073, 2.503221292
H, 0.0437223324, 2.1848099075, 1.211033786
H, 0.0437223337, 2.1848099069, −1.2110337873
H, 0.3802444846, 0.0171777062, −2.5032212919
H, 0.8199460897, −2.1143749021, −1.2526227442
Be, −1.4811721037, −1.80316123, −0.0000000005
H, −1.4112558474, −3.1719607293, −0.0000000001
H, −2.3018476673, −0.7236816858, −0.0000000012
Br, 0.2638909508, 4.7215260437, −0.0000000011

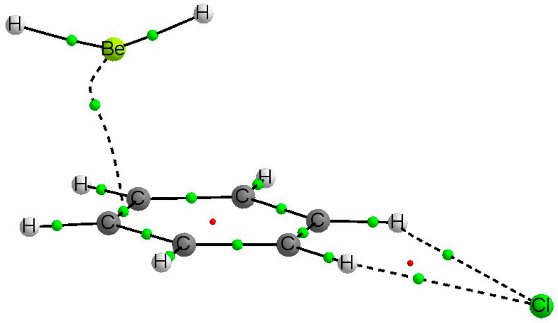

Cl^-:C_6H_6:BeH_2 MP2 = −707.10826090 NIMAG = 0
C, 0.611075753, −1.177151914, 0.7091039342
C, 0.3871789153, 0.0299684865, 1.4077934597
C, 0.1967698381, 1.2351691655, 0.7039847744
C, 0.1967698388, 1.2351691652, −0.7039847751
C, 0.3871789168, 0.0299684858, −1.4077934596
C, 0.6110757537, −1.1771519143, −0.7091039333
H, 0.8133964087, −2.1065595033, 1.2526929799
H, 0.3822096988, 0.0264932419, 2.5034490736
H, 0.0574851042, 2.2009483613, 1.2038946945
H, 0.0574851055, 2.2009483607, −1.2038946958
H, 0.3822097015, 0.0264932407, −2.5034490735
H, 0.8133964101, −2.1065595039, −1.2526929783
Be, −1.477440505, −1.7912996614, −0.0000000005
H, −1.4123400312, −3.1612012431, −0.0000000002
H, −2.3033351226, −0.7152816333, −0.0000000012
Cl, 0.2294762716, 4.5644305654, −0.0000000011

Table S1. *Cont.*

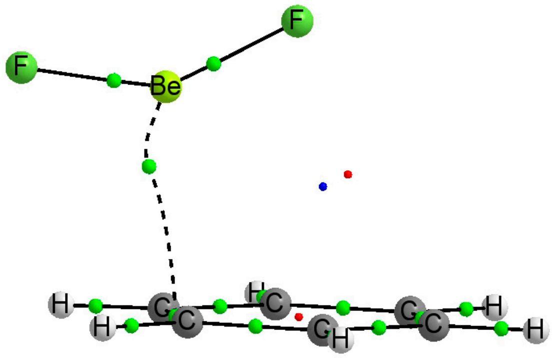

C_6H_6:BeF_2 MP2 = −445.70937508 NIMAG = 0
C, 0.7166707495, −0.8217203662, 0.7093006754
C, 0.180940058, 0.2787240688, 1.4121529707
C, −0.3531693924, 1.369658695, 0.7038274062
C, −0.3531693924, 1.369658695, −0.7038274062
C, 0.180940058, 0.2787240688, −1.4121529707
C, 0.7166707495, −0.8217203662, −0.7093006754
H, 1.1540876782, −1.6672051414, 1.249520047
H, 0.1782936139, 0.2773802013, 2.5064693049
H, −0.7788101668, 2.2174729056, 1.2492279378
H, −0.7788101668, 2.2174729056, −1.2492279378
H, 0.1782936139, 0.2773802013, −2.5064693049
H, 1.1540876782, −1.6672051414, −1.249520047
Be, −1.1978989165, −1.9328295552, 0.
F, −0.7199160246, −3.2949810578, 0.
F, −2.3456180756, −1.0664263966, 0.

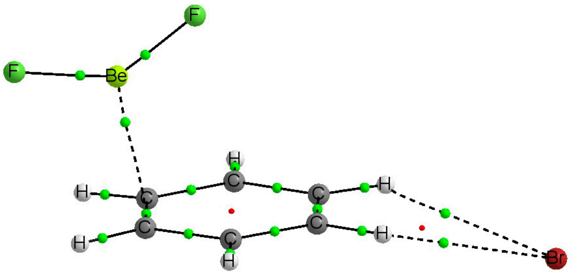

Br^-:C_6H_6:BeF_2 MP2 = −3018.34274853 NIMAG = 0
C, 0.5720275988, −1.162078929, 0.7109089689
C, 0.3636301155, 0.0486685803, 1.4103150182
C, 0.1781976206, 1.2508489409, 0.7050238187
C, 0.1781976206, 1.2508489409, −0.7050238197
C, 0.3636301155, 0.0486685803, −1.4103150192
C, 0.5720275988, −1.162078929, −0.7109089699
H, 0.7763216428, −2.092889539, 1.2509281188
H, 0.3625823261, 0.0467591397, 2.5054716337
H, 0.0519084187, 2.2151592623, 1.2101792984
H, 0.0519084187, 2.2151592623, −1.2101792994
H, 0.3625823261, 0.0467591397, −2.5054716347
H, 0.7763216428, −2.092889539, −1.2509281198
Be, −1.401943658, −1.8451798009, −0.0000000005
F, −1.2696511506, −3.3006638613, −0.0000000005
F, −2.4595153618, −0.8624628068, −0.0000000005
Br, 0.4543667674, 4.709755265, −0.0000000005

Table S1. *Cont.*

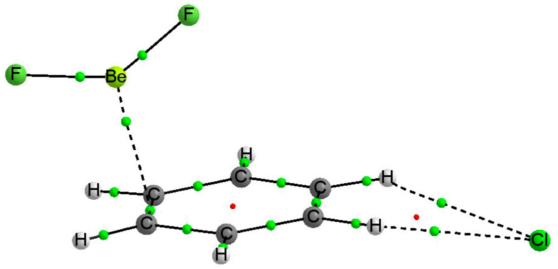

Cl⁻:C_6H_6:BeF_2 MP2 = −905.45747792 NIMAG = 0
C, 0.5679499686, −1.1541837563, 0.7110385747
C, 0.3678982464, 0.0582859559, 1.4104763758
C, 0.1919784646, 1.2619184797, 0.7051743095
C, 0.1919784646, 1.2619184797, −0.7051743103
C, 0.3678982464, 0.0582859559, −1.4104763766
C, 0.5679499686, −1.1541837563, −0.7110385755
H, 0.7662870326, −2.0864945364, 1.2508408353
H, 0.3663754206, 0.0555859852, 2.5057367792
H, 0.0738269764, 2.2316692997, 1.2028262937
H, 0.0738269764, 2.2316692997, −1.2028262945
H, 0.3663754206, 0.0555859852, −2.5057367799
H, 0.7662870326, −2.0864945364, −1.250840836
Be, −1.4023168135, −1.8330114879, 0.
F, −1.2758842719, −3.2899089973, −0.0000000004
F, −2.4636976162, −0.8536239067, −0.0000000004
Cl, 0.4058585485, 4.5573652369, −0.0000000004

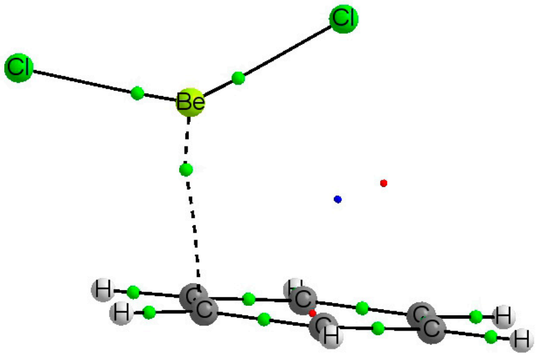

C_6H_6:$BeCl_2$ MP2 = −1165.69066708 NIMAG = 0
C, 0.7027531187, −0.8014838975, 0.7105341951
C, 0.1915448151, 0.3114543473, 1.4129667548
C, −0.3178392441, 1.4133996568, 0.7042289051
C, −0.3178392441, 1.4133996568, −0.7042289051
C, 0.1915448151, 0.3114543473, −1.4129667548
C, 0.7027531187, −0.8014838975, −0.7105341951
H, 1.142132852, −1.6453679079, 1.2519003756
H, 0.1888127275, 0.3099040859, 2.50732886
H, −0.7258920843, 2.2701193857, 1.2492078725
H, −0.7258920843, 2.2701193857, −1.2492078725
H, 0.1888127275, 0.3099040859, −2.50732886
H, 1.142132852, −1.6453679079, −1.2519003756
Be, −1.141695336, −1.9669981768, 0.
Cl, −0.5641482233, −3.7464465493, 0.
Cl, −2.7245887659, −0.9882229095, 0.

Table S1. *Cont.*

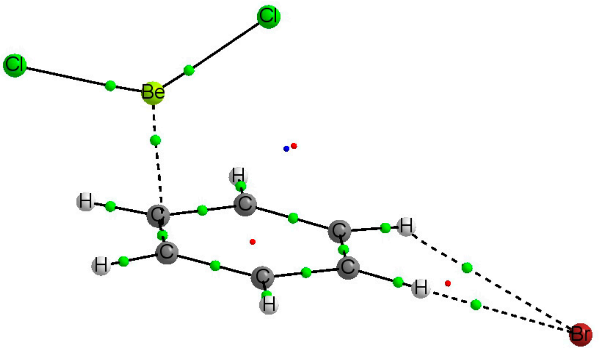

Br⁻:C_6H_6:$BeCl_2$ MP2 = −3738.32736513 NIMAG = 0
C, 0.2462707594, −2.4668938711, 0.7125263697
C, 0.2503060592, −1.2372008349, 1.4109852598
C, 0.2802691664, −0.021318536, 0.7051763991
C, 0.2802691664, −0.021318536, −0.7051763991
C, 0.2503060592, −1.2372008349, −1.4109852598
C, 0.2462707594, −2.4668938711, −0.7125263697
H, 0.3094095061, −3.4170219407, 1.253826336
H, 0.2458804725, −1.239293931, 2.5060985854
H, 0.3277443299, 0.9507571156, 1.2092772003
H, 0.3277443299, 0.9507571156, −1.2092772003
H, 0.2458804725, −1.239293931, −2.5060985854
H, 0.3094095061, −3.4170219407, −1.253826336
Be, −1.7663301992, −2.8127362721, 0.
Cl, −2.0227005771, −4.6993106303, 0.
Cl, −2.9758611022, −1.3825599629, 0.
Br, 1.2581352555, 3.2726088799, 0.

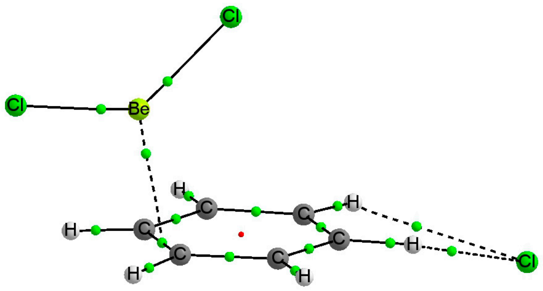

Cl⁻:C_6H_6:$BeCl_2$ MP2 = −1625.44225034 NIMAG = 0
C, 0.5153501772, −1.6793031196, 0.7126802469
C, 0.509107463, −0.4493149224, 1.4111056511
C, 0.5298596868, 0.7668739951, 0.7053200961
C, 0.5298596868, 0.7668739951, −0.7053200961
C, 0.509107463, −0.4493149224, −1.4111056511
C, 0.5153501772, −1.6793031196, −0.7126802469
H, 0.5872571489, −2.6289614866, 1.2538370174
H, 0.504256828, −0.4521486724, 2.5063142824
H, 0.5715441462, 1.7436364666, 1.2018844631
H, 0.5715441462, 1.7436364666, −1.2018844631
H, 0.504256828, −0.4521486724, −2.5063142824
H, 0.5872571489, −2.6289614866, −1.2538370174
Be, −1.4876935801, −2.0484364068, 0.
Cl, −1.7256923505, −3.939047462, 0.
Cl, −2.7198318552, −0.6369351834, 0.
Cl, 1.354993898, 3.9314305306, 0.

Table S2. Molecular graphs, MP2 energy (Hartree), Number of Imaginary Frequencies (NIMAG) and Cartesian coordinates (Å) of C_6F_6, C_6F_6: X^-, BeR_2:C_6F_6 and BeR_2:C_6F_6:X^- systems.

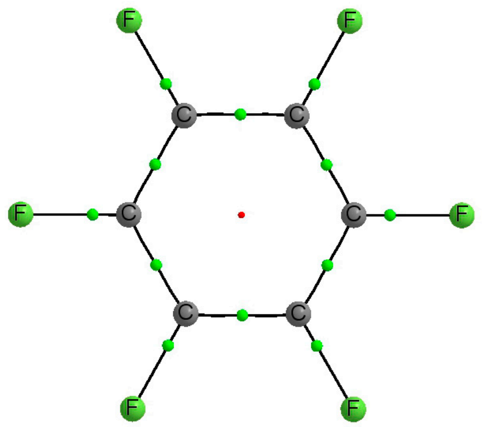

C_6F_6 MP2 = −825.83972849 NIMAG = 0
C, 0.911266765, −0.759597649, 0.7005947318
C, 0.264231777, 0.2670822926, 1.4009370781
C, −0.3829084153, 1.2937445447, 0.7006019122
C, −0.382908446, 1.2937445939, −0.7006018115
C, 0.2642317155, 0.267082391, −1.4009370779
C, 0.9112667343, −0.7595975998, −0.7005948322
F, 1.5328927352, −1.7454932278, 1.3737129125
F, 0.2648272056, 0.2674178633, 2.7471162288
F, −1.0035359371, 2.2802746733, 1.3739028022
F, −1.0035359974, 2.2802747698, −1.3739026049
F, 0.2648270851, 0.2674180564, −2.7471162286
F, 1.5328926749, −1.7454931313, −1.3737131095

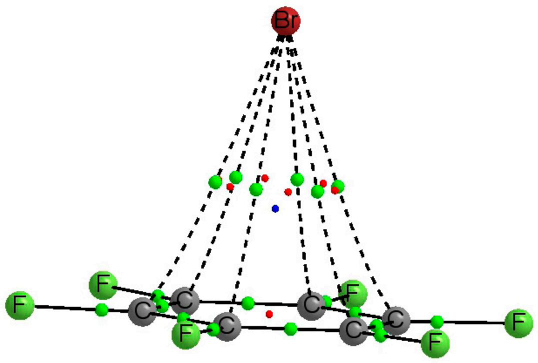

C_6F_6:Br^- MP2 = −3398.47408565 NIMAG = 0
C, 1.3969839, 0.0000000, 0.0000000
C, 0.6984919, −1.2098235, 0.0000000
C, −0.6984919, −1.2098235, 0.0000000
C, −1.3969839, 0.0000000, 0.0000000
C, −0.6984919, 1.2098235, 0.0000000
C, 0.6984919, 1.2098235, 0.0000000
F, 2.7485503, 0.0000000, −0.0202356
F, 1.3742752, −2.3803144, −0.0202356
F, −1.3742752, −2.3803144, −0.0202356
F, −2.7485503, 0.0000000, −0.0202356
F, −1.3742752, 2.3803144, −0.0202356
F, 1.3742752, 2.3803144, −0.0202356
Br, 0.0000000, 0.0000000, −3.2133507

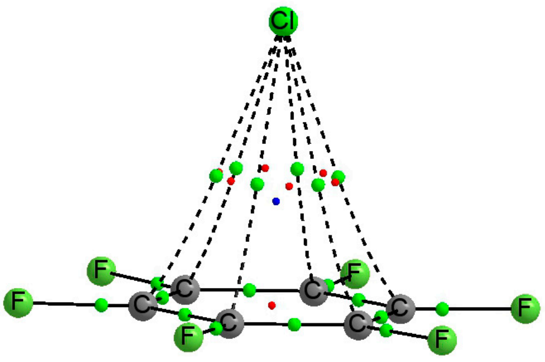

C_6F_6:Cl^- MP2 = −1285.58803712 NIMAG = 0
C, 1.3966363, 0.0000000, 0.0000000
C, 0.6983182, −1.2095225, 0.0000000
C, −0.6983182, −1.2095225, 0.0000000
C, −1.3966363, 0.0000000, 0.0000000
C, −0.6983182, 1.2095225, 0.0000000
C, 0.6983182, 1.2095225, 0.0000000
F, 2.7489492, 0.0000000, −0.0197052
F, 1.3744746, −2.3806599, −0.0197052
F, −1.3744746, −2.3806599, −0.0197052
F, −2.7489492, 0.0000000, −0.0197052
F, −1.3744746, 2.3806599, −0.0197052
F, 1.3744746, 2.3806599, −0.0197052
Cl, 0.0000000, 0.0000000, −3.0639319

Table S2. *Cont.*

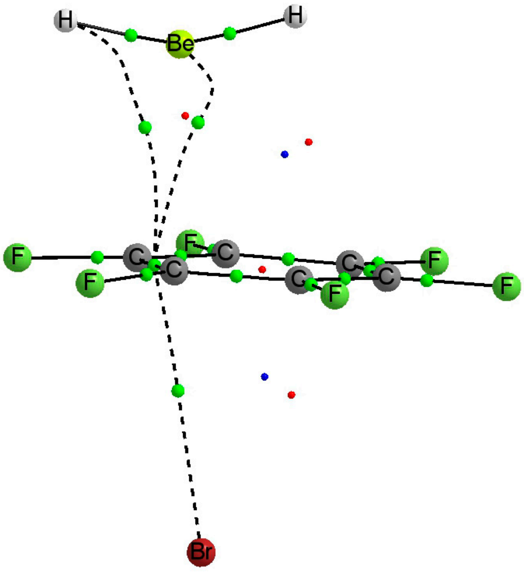

Br$^-$:C$_6$F$_6$:BeH$_2$ MP2 = −3414.30430516 NIMAG = 0
C, 0.1325222684, −1.1116544707, 0.702815867
C, 0.0764975044, 0.1043032811, 1.3980356454
C, 0.0392036586, 1.3131876001, 0.6976905227
C, 0.0392036586, 1.3131876001, −0.6976905227
C, 0.0764975044, 0.1043032811, −1.3980356454
C, 0.1325222684, −1.1116544707, −0.702815867
F, 0.2786965048, −2.2633028905, 1.376666024
F, 0.1041717192, 0.1068397837, 2.7445041517
F, −0.0087059004, 2.4797332342, 1.372548301
F, −0.0087059004, 2.4797332342, −1.372548301
F, 0.1041717192, 0.1068397837, −2.7445041517
F, 0.2786965048, −2.2633028905, −1.376666024
Be, −2.2805353226, −1.0724085638, 0.
H, −2.4485183431, −2.4139684674, 0.
H, −2.676776158, 0.2200059575, 0.
Br, 3.2277988126, −0.283545328, 0.

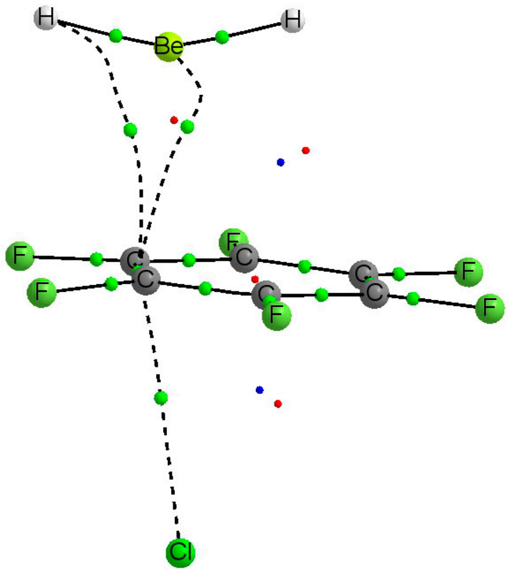

Cl$^-$:C$_6$F$_6$:BeH$_2$ MP2 = −1301.41866829 NIMAG = 0
C, 0.1445888962, −1.1051154971, 0.7027465033
C, 0.0824670651, 0.110441153, 1.3977432539
C, 0.0421894986, 1.3190078102, 0.6976179087
C, 0.0421894986, 1.3190078102, −0.6976179087
C, 0.0824670651, 0.110441153, −1.3977432539
C, 0.1445888962, −1.1051154971, −0.7027465033
F, 0.298036247, −2.2553943846, 1.3765973198
F, 0.1101595374, 0.1132640697, 2.7447505612
F, −0.0126336511, 2.4861760477, 1.3726962865
F, −0.0126336511, 2.4861760477, −1.3726962865
F, 0.1101595374, 0.1132640697, −2.7447505612
F, 0.298036247, −2.2553943846, −1.3765973198
Be, −2.251188797, −1.0764293964, 0.
H, −2.4264909177, −2.4180009492, 0.
H, −2.6640428726, 0.2117468799, 0.
Cl, 3.0788479152, −0.3457782566, 0.

Table S2. *Cont.*

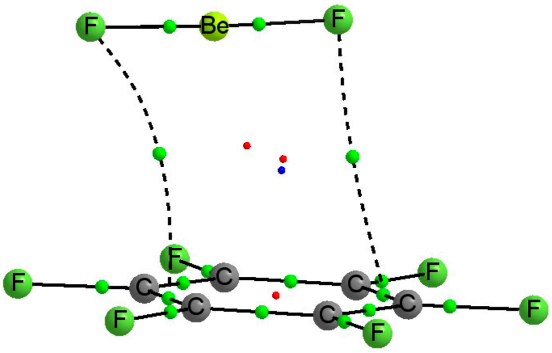

C_6F_6:BeF_2 MP2 = −1040.00401005 NIMAG = 0
C, 0.8413256116, −0.9062017651, 0.6023779626
C, 0.4637216482, 0.2178852601, 1.3546486432
C, 0.1419319966, 1.4204558315, 0.7094893294
C, 0.1909799019, 1.5022060413, −0.687686493
C, 0.5646390283, 0.3825414195, −1.441299043
C, 0.8869090959, −0.8212163145, −0.7986087982
F, 1.194536075, −2.0429626217, 1.2203422276
F, 0.4253419748, 0.1437383079, 2.6978675515
F, −0.2324316835, 2.4894664003, 1.4334722934
F, −0.1434566924, 2.6464290677, −1.3055768469
F, 0.5962837279, 0.4545533931, −2.7832611239
F, 1.2550205345, −1.8935514038, −1.5237543341
Be, −2.0853864971, −1.2210304403, 0.1788243737
F, −1.780716965, −2.4426873601, 0.8292303594
F, −2.4444037567, −0.012161816, −0.4760661018

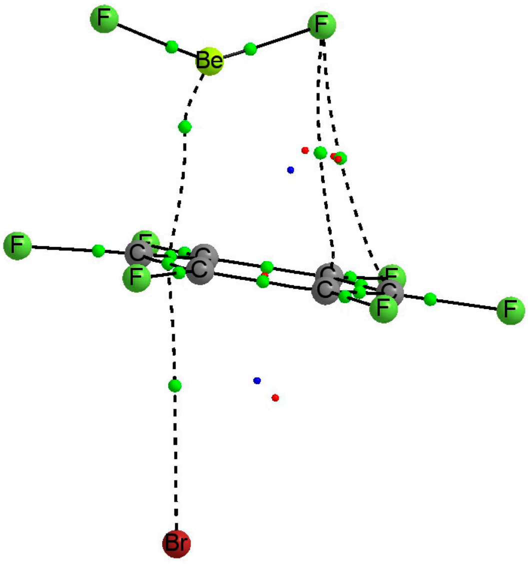

Br^-:C_6F_6:BeF_2 MP2 = −3612.65022072 NIMAG = 0
C, 0.1175083571, −1.1030326408, 0.704912836
C, 0.0816924121, 0.1152730641, 1.4006026026
C, 0.0399785231, 1.3210574958, 0.6985665022
C, 0.0399785231, 1.3210574958, −0.6985665022
C, 0.0816924121, 0.1152730641, −1.4006026026
C, 0.1175083571, −1.1030326408, −0.704912836
F, 0.2712721807, −2.2535587444, 1.3739535881
F, 0.1090021549, 0.1168356143, 2.7451182064
F, −0.0154584213, 2.4871618492, 1.3692553835
F, −0.0154584213, 2.4871618492, −1.3692553835
F, 0.1090021549, 0.1168356143, −2.7451182064
F, 0.2712721807, −2.2535587444, −1.3739535881
Be, −2.1518988504, −1.0683494636, 0.
F, −2.4339913316, −2.4787349453, 0.
F, −2.7407676204, 0.2480277751, 0.
Br, 3.1854078613, −0.3601199714, 0.

Table S2. *Cont.*

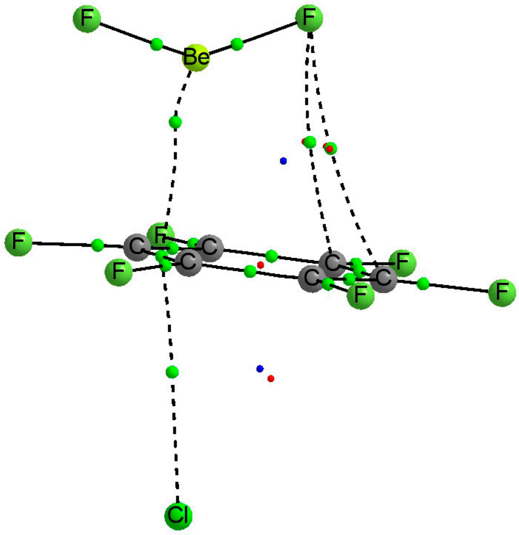

$Cl^-:C_6F_6:BeF_2$ MP2 = −1499.76478931 NIMAG = 0
C, 0.1304599769, −1.0981975014, 0.7048033292
C, 0.088693543, 0.1197124033, 1.4003316349
C, 0.0448868971, 1.3251720136, 0.698525503
C, 0.0448868971, 1.3251720136, −0.698525503
C, 0.088693543, 0.1197124033, −1.4003316349
C, 0.1304599769, −1.0981975014, −0.7048033292
F, 0.2896186333, −2.2476259334, 1.3739267917
F, 0.1158482911, 0.1215292413, 2.7454292226
F, −0.0169578944, 2.4918652088, 1.3694522828
F, −0.0169578944, 2.4918652088, −1.3694522828
F, 0.1158482911, 0.1215292413, −2.7454292226
F, 0.2896186333, −2.2476259334, −1.3739267917
Be, −2.1304688645, −1.0703623802, 0.
F, −2.4144496363, −2.4813708572, 0.
F, −2.7330534999, 0.24080587, 0.
Cl, 3.0396136126, −0.405686823, 0.

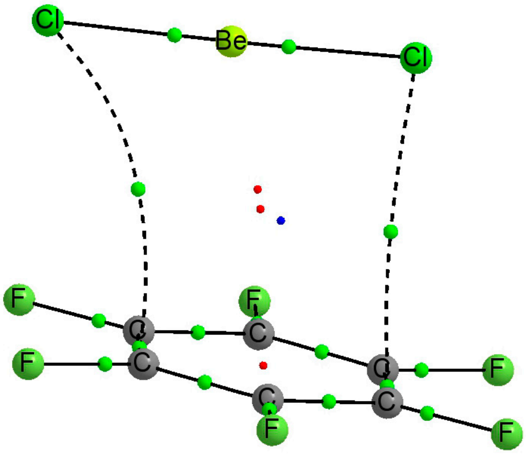

$C_6F_6:BeCl_2$ MP2 = −1759.98660426 NIMAG = 0
C, 0.9645201323, −0.7349155382, 0.7019134407
C, 0.3123253396, 0.2908251057, 1.4010614028
C, −0.3357499052, 1.3163936209, 0.7001087293
C, −0.3357499052, 1.3163936209, −0.7001087296
C, 0.3123253396, 0.2908251057, −1.401061403
C, 0.9645201323, −0.7349155382, −0.7019134409
F, 1.6092532906, −1.703857947, 1.3740803599
F, 0.3023081338, 0.2847141677, 2.746919026
F, −0.9823339387, 2.284490655, 1.372184801
F, −0.9823339387, 2.284490655, −1.3721848012
F, 0.3023081338, 0.2847141677, −2.7469190263
F, 1.6092532906, −1.703857947, −1.3740803602
Be, −1.9228060303, −2.1447975785, 0.
Cl, −0.8844943869, −3.6335273126, 0.
Cl, −3.000334999, −0.6795467166, 0.

Table S2. *Cont.*

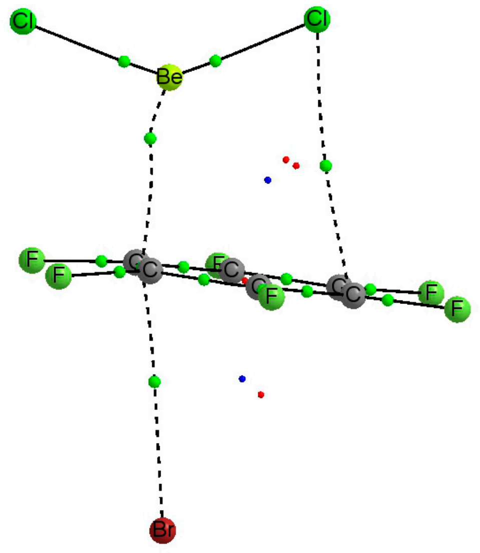

Br^-:C_6F_6:$BeCl_2$ MP2 = −4332.6331655 NIMAG = 0
C, 0.1283133987, −1.0853669684, 0.7064373673
C, 0.1052101693, 0.1350343736, 1.4014736923
C, 0.082866932, 1.3405356251, 0.698911173
C, 0.082866932, 1.3405356251, −0.698911173
C, 0.1052101693, 0.1350343736, −1.4014736923
C, 0.1283133987, −1.0853669684, −0.7064373673
F, 0.3094124745, −2.2299383801, 1.3752078145
F, 0.1310269603, 0.1364877611, 2.7442515454
F, 0.0379973611, 2.5065099727, 1.3676376433
F, 0.0379973611, 2.5065099727, −1.3676376433
F, 0.1310269603, 0.1364877611, −2.7442515454
F, 0.3094124745, −2.2299383801, −1.3752078145
Be, −2.1244769969, −1.1047308233, 0.
Cl, −2.5334123442, −2.9202010207, 0.
Cl, −3.0423786912, 0.5236600905, 0.
Br, 3.1773539172, −0.3969563438, 0.

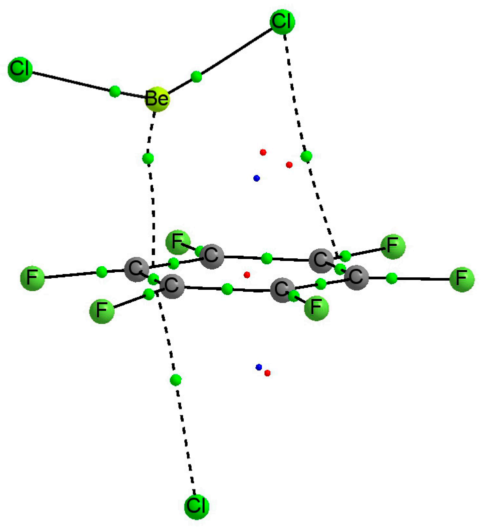

Cl^-:C_6F_6:$BeCl_2$ MP2 = −2219.7479135 NIMAG = 0
C, 0.1420374244, −1.0806260026, 0.706333468
C, 0.1118848184, 0.13947003, 1.4011786125
C, 0.0877376737, 1.3447296534, 0.6989006523
C, 0.0877376737, 1.3447296534, −0.6989006523
C, 0.1118848184, 0.13947003, −1.4011786125
C, 0.1420374244, −1.0806260026, −0.706333468
F, 0.3284751274, −2.2238569548, 1.3750119752
F, 0.1367960727, 0.141077959, 2.7445470619
F, 0.0359995096, 2.5112623732, 1.3678500823
F, 0.0359995096, 2.5112623732, −1.3678500823
F, 0.1367960727, 0.141077959, −2.7445470619
F, 0.3284751274, −2.2238569548, −1.3750119752
Be, −2.1003024349, −1.1050845722, 0.
Cl, −2.5142391854, −2.9213393894, 0.
Cl, −3.0348529958, 0.515860785, 0.
Cl, 3.0302738505, −0.4452542688, 0.

Table S3. HOMA aromaticity index.

System	Homa Index	System	HOMA index
C_6H_6	1.00	C_6F_6	0.99
BeH_2:C_6H_6	1.00	BeF_2:C_6F_6	0.99
BeF_2:C_6H_6	0.99	BeF_2:C_6F_6	0.99
$BeCl_2$:C_6H_6	0.99	$BeCl_2$:C_6F_6	0.99
C_6H_6:Br^-	1.00	C_6F_6:Br^-	0.97
C_6H_6:Cl^-	1.00	C_6F_6:Cl^-	0.97
BeH_2:C_6H_6:Br^-	0.99	BeH_2:C_6F_6:Br^-	0.98
BeH_2:C_6H_6:Cl^-	0.99	BeH_2:C_6F_6:Cl^-	0.98
BeF_2:C_6H_6:Br^-	0.99	BeF_2:C_6F_6:Br^-	0.98
BeF_2:C_6H_6:Cl^-	0.99	BeF_2:C_6F_6:Cl^-	0.98
$BeCl_2$:C_6H_6 :Br^-	0.98	$BeCl_2$:C_6F_6:Br^-	0.98
$BeCl_2$:C_6H_6:Cl^-	0.98	$BeCl_2$:C_6F_6:Cl^-	0.98
